# Action Observation and Motor Imagery: Innovative Cognitive Tools in the Rehabilitation of Parkinson's Disease

**DOI:** 10.1155/2015/124214

**Published:** 2015-10-01

**Authors:** Giovanni Abbruzzese, Laura Avanzino, Roberta Marchese, Elisa Pelosin

**Affiliations:** ^1^Department of Neuroscience, Rehabilitation, Ophthalmology, Genetics and Maternal Child Health, University of Genoa, 16132 Genoa, Italy; ^2^Department of Experimental Medicine, Section of Human Physiology, University of Genoa, 16132 Genoa, Italy

## Abstract

Parkinson's disease (PD) is characterized by a progressive impairment of motor skills with deterioration of autonomy in daily living activities. Physiotherapy is regarded as an adjuvant to pharmacological and neurosurgical treatment and may provide small and short-lasting clinical benefits in PD patients. However, the development of innovative rehabilitation approaches with greater long-term efficacy is a major unmet need. Motor imagery (MI) and action observation (AO) have been recently proposed as a promising rehabilitation tool. MI is the ability to imagine a movement without actual performance (or muscle activation). The same cortical-subcortical network active during motor execution is engaged in MI. The physiological basis of AO is represented by the activation of the “mirror neuron system.” Both MI and AO are involved in motor learning and can induce improvements of motor performance, possibly mediated by the development of plastic changes in the motor cortex. The review of available evidences indicated that MI ability and AO feasibility are substantially preserved in PD subjects. A few preliminary studies suggested the possibility of using MI and AO as parts of rehabilitation protocols for PD patients.

## 1. Introduction

Parkinson's disease (PD) is a complex neurodegenerative disorder characterized by motor and nonmotor symptoms. Since no known cure exists, the management of PD is traditionally based on symptomatic treatment with drug therapy (levodopa being considered the “gold standard”) or with neurosurgical approaches (Deep Brain Stimulation, DBS). However, even with optimal medical or surgical management, patients with PD still experience a progressive deterioration of their autonomy with increasing difficulties in daily living activities and in various aspects of mobility such as gait, transfers, balance, and posture. For this reason, there has been increasing recourse to the inclusion of rehabilitation therapies as an adjuvant to pharmacological and neurosurgical treatment with the aim of maximizing functional ability and minimizing secondary complications.

A recent meta-analysis of physiotherapy interventions [1] provided evidence of short-term, small but significant and clinically important benefits for walking speed and balance in PD patients. However, formal comparison of different techniques could not be performed and there was insufficient evidence to support one specific physiotherapy intervention [2]. The latter reviews pointed out the need for more adequate trials and for the development of innovative approaches demonstrating a longer-term efficacy and better cost-effectiveness of physiotherapy in PD. Traditionally, physiotherapy was based on physical practice to improve motor abilities (such as muscular strength, gait, or coordination); however, the new guidelines highlighted that physiotherapy for PD needs to maximise quality of movement and functional independence by means of a tailored intervention linked to the stage of the disease progression.

With regard to physiotherapy interventions, several approaches aim to teach patients using compensatory attentional/cognitive strategies that may rely on the recruitment of alternative motor circuits. Indeed, it has been demonstrated that both cueing strategies (based on the use of external stimuli associated with the initiation and facilitation of a motor activity) and attentional strategies (such as instructions which rely on cognitive mechanisms of motor control and are internally generated) are able to improve walking performance by using alternative pathways unaffected by PD [3]. In this sense, motor imagery (MI) and action observation (AO) are two training techniques that have recently gained attention as a promising rehabilitation tool for patients with neurological disorders [4–6].

The aim of this perspective review was to show that both motor imagery (MI) and action observation (AO) represent two innovative rehabilitation approaches that are feasible in Parkinson's disease (PD) and potentially able to induce significant benefits. Here we briefly summarized the basic mechanisms underlying MI and AO, their role in motor learning, and possible abnormalities in patients with PD. Further, we reviewed the available evidences supporting the use of MI and AO in the rehabilitation of Parkinsonian subjects.

## 2. Motor Imagery and Motor Learning

**Figure 1:**
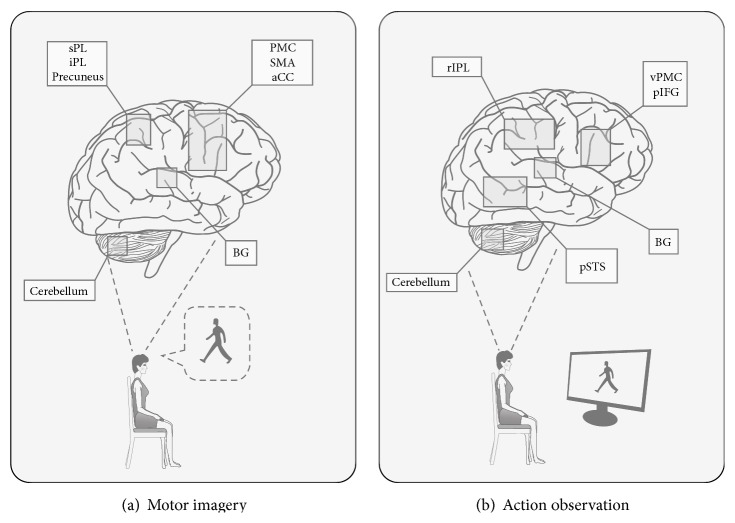
The human brain activity during motor imagery (a) and action observation (b). (a) shows brain areas activated during kinesthetic and visual motor imagery. The pattern of activity includes the following regions: ventral and dorsal part of the premotor cortex (PMC); supplementary motor area (SMA); anterior Cingulate Cortex (aCC); superior Parietal Lobule (sPL) and inferior Parietal Lobule (iPL); precuneus; basal ganglia (BG); and cerebellum. (b) shows the complex brain network (“mirror neuron system”) involved in action observation: ventral premotor cortex (vPMC), posterior part of the Inferior Frontal Gyrus (pIFG), rostral part of the Inferior Parietal Lobule (rIPL), and posterior Superior Temporal Sulcus (pSTS).

Motor imagery (MI) is a cognitive process in which a subject imagines that he is performing a movement without actually doing it and without even tensing the muscles (Figure 1(a)). It is a complex, self-generated, dynamic state during which the representation of a specific motor action is internally activated without any motor output [7, 8]. MI has been categorized as external (visual) and internal (kinesthetic) and the perspective the person uses to imagine can be either the first or the third person. The “first-person” perspective is related either to the person's view of the imagery contents or to its kinesthetic sensation, while the “third-person” perspective is the visual imagery of scenes outside the person.

Jeannerod and Decety [9] suggested that MI would represent the result of conscious access to the intention to move, suggesting that conscious motor imagery and unconscious motor preparation are likely to share common mechanisms. Indeed, a large body of evidence suggests that imagined and executed actions share the same neural structures recruiting overlapping brain regions (i.e., premotor cortex, anterior cingulate, inferior Parietal Lobule, and cerebellum) [10, 11], although MI is thought to reflect mainly the process of movement preparation, with reduced involvement of end-stage movement execution related processes [12, 13].

Besides the overlap in neural activation between imagery and execution there are also similarities in the behavioural domain. For instance, the time to complete an imagined movement is known to be similar to the time needed for actual execution of that movement [14]. This phenomenon is known as mental isochrony. Decety et al. [15] studied subjects who were instructed to either actually perform or mentally simulate a leg exercise. Heart rate and respiration rate were measured in both conditions. The results showed that the heart rate and respiration rate began to increase not only during actual exercise but also in the mental condition where no work at all was produced. These findings have led to a theoretical position termed the “simulation hypothesis” suggesting that movement execution and MI are driven by the same basic mechanisms [16].

On the basis of all these data, it is reasonable to think that, like motor execution, MI training can induce improvements in motor performance and thus in motor learning processes.

Pascual-Leone et al. [17] showed that during 5 days of training of a musical performance both MI and motor execution resulted in an increase in performance although the motor execution group outperformed the motor imagery group. Interestingly, the MI group demonstrated the same training effect as the motor execution group after only one additional execution session. Further, MI has been demonstrated to modify the actual speed of execution of body movements [18]; the authors investigated the effect of changing MI speed on actual movement duration over a 3-week training period. Participants mentally performed a series of body movements faster or slower than their actual execution speeds. The fast MI group's actual times decreased on subsequent performance. The effect of MI on actual speed execution supports the ideomotor theory because anticipation of sensory consequences of actions is mentally represented. The beneficial effects of mental practice on the physical performance have been suggested to rely on the close temporal association between motor rehearsal and actual performance. In the same vein, Avanzino et al. [19] showed that motor imagery is able to improve the performance of repetitive finger opposition movements more than the motor practice alone. Further, when subjects performed MI, they speeded up the movement by modifying different kinematic aspects of finger opposition movements, thus suggesting that motor imagery was able to significantly improve movement speed by inducing a modification in the specific motor program.

At the basis of motor performance improvement induced by MI is that the same cortical-subcortical network, active during motor execution, is engaged in MI [9, 10]. In accordance with that, it has been demonstrated that motor imagery training leads to the development of neuroplasticity in the primary motor cortex (M1), as it affected transcranial magnetic stimulation induced plasticity in M1 [20].

## 3. Motor Imagery in Parkinson's Disease

The ability of people with PD to efficiently imagine movements is still controversial. Abnormal performance on motor imagery tasks was initially suggested in patients with PD using different approaches, including behavioural, electrophysiological (transcranial magnetic stimulation, TMS, and movement-related potentials, MRPs), and functional imaging studies.

Tremblay et al. [21] investigated the facilitation of motor evoked potentials (MEPs) to TMS during action imagination. Corticomotor facilitation was defective in medicated PD patients thus supporting the hypothesis of an impaired motor preparation associated with basal ganglia dysfunction in PD. Cunnington et al. [22] reported that MRPs, recorded during motor imagery of an externally paced sequential button-pressing task, were present but significantly reduced in amplitude and abnormally prolonged in PD. However, the preparatory-phase associated with motor imagery was mainly impaired in patients with more severe Parkinsonian symptoms and not in early-stage PD. Consistent with this finding, a PET study by the same authors [23] showed that imagined movements of PD patients in the “off” condition were associated with reduced activation of specific cortical areas (including the anterior cingulate and the right dorsolateral prefrontal cortex, DLPFC) but also with compensatory activation of additional areas (ipsilateral premotor and inferior parietal cortex).

Although brain activation during MI is abnormal in Parkinsonian subjects [24], the possible occurrence of compensation during MI was documented in PD using fMRI [25]: in strongly lateralized PD patients, MI of the most-affected hand recruited additional resources in extrastriate visual areas (and their connections with premotor cortex). Conversely, the inhibition by repetitive TMS (theta burst stimulation) of the right extrastriate body area abolished in PD patients but not in healthy subjects the compensatory effect on MI [26].

These studies basically highlighted functional changes in the activation of corticostriatal circuits in relation to the imagery of motor tasks in PD subjects, further supporting general abnormalities of motor planning in this condition. Indeed, the motor corticostriatal circuit seems to be engaged during motor imagery. In PD patients implanted for DBS it has been shown that imagination of a simple, repetitive movement significantly reduced the neuronal firing rate of GPi neurons [27]. Similarly, oscillatory beta activity in the region of the subthalamic nucleus (STN) was modulated to the same extent during motor execution and imagination [28]. Stimulation of the STN was also demonstrated to change PET activation during actual or imagined movements in PD [29].

Altogether, experimental results support preserved MI ability in PD, but with different patterns of cerebral activity [30]. In keeping with this hypothesis, recent contributions suggested a substantially normal efficiency of MI processes in PD. Heremans et al. [31] used an extensive imagery ability assessment battery to test 14 PD patients and 14 normal subjects. They found that physical execution was slowed to the same extent as MI, indicating that the slowness of MI reflects the bradykinesia inherent to PD rather than an inability to correctly perform it. These authors [32] also investigated whether the quality of MI could be improved by external cueing. The presence of visual cues significantly reduced the patients' bradykinesia during MI and increased their imagery vividness.

The influence of pharmacological (levodopa) treatment was also investigated: the vividness of MI was not different between the “on” and “off” conditions or between PD and controls [33]. These results suggest that although levodopa has been suggested to normalize brain activity in several cortical areas (including the supplementary motor area), PD patients are able to imagine similarly to older adults when both “on” and “off” anti-Parkinson medication. A recent study by Maillet et al. [34] showed that “kinesthetic” motor imagery abilities are preserved in PD patients and can be further improved by training.

Finally, we recently used MI to investigate time processing abilities (time estimation and reproduction) in PD patients [35]: a similar behaviour was observed during imagery task and in the execution task. Likewise, Conson et al. [36] demonstrated a parallel impairment between motor and mental simulation mechanisms in PD patients. To further support the ability of Parkinsonian patients to mentally simulate physical activities, MI was also used during fMRI to investigate locomotion related brain activity in PD [37, 38].

We may conclude that MI ability is substantially preserved in PD subjects (particularly in the mid and early stage), although it might be “slow” in comparison to healthy controls. In particular, it is likely that PD patients may use a compensatory “third-person” strategy rather than using MI from a “first-person” perspective. The studies, therefore, support the possible use and implementation of motor imagery training in the rehabilitation of patients with PD.

Although MI ability was extensively investigated in PD, very few studies have tested the possibility of using MI as part of rehabilitation protocols for PD patients (see Table 1).

The combination of MI and physical practice was compared to physical therapy alone in a randomized-controlled (RC) trial [39]. Both groups practiced callisthenic exercises, functional task, and relaxation exercises. However, the experimental group (treated with both imagery and real practice) exhibited faster performance of motor sequences (reduced bradykinesia). Interestingly, the implementation of MI allowed higher gains in the mental subsets of the Unified Parkinson's Disease Rating Scale (UPDRS).

On the other hand, Braun et al. [40] compared mental practice with relaxation embedded in standard physiotherapy and did not find any significant difference in walking performance and related outcome measures. Finally, a recent RC single-blinded trial [41] investigated autogenetic training (AT) based on visual imagery. When used as an adjunct to physical therapy, AT proved more effective than physical therapy alone in improving motor performances (UPDRS motor section) in 66 PD patients.

## 4. Action Observation and Motor Learning

It is widely accepted that the observation of actions performed by others activates in the brain the same neural structures used for the actual execution of the same actions. The neurophysiological basis of “action observation” (AO) (Figure 1(b)) is represented by the discovery of mirror neurons in the monkey cerebral cortex [42, 43] that discharge during both the execution of goal-directed actions and the observation of other individuals performing similar actions. The definition of “mirror neuron system” (MNS) comprises the cerebral areas containing mirror neurons and evidences with the use of TMS and functional imaging (fMRI) suggested that an MNS is also present in the human brain [44].

In humans during AO the excitability of the motor cortex is enhanced [45] and the 15–25 Hz EEG activity is suppressed [46]. AO, therefore, is able to recruit specific areas in the frontal and parietal lobes similarly to what happens during motor execution. Such effect is maximal when the observed actions are familiar and belong to the motor repertoire of the observers. The MNS has been shown to be also involved in “imitation” within a circuit involving the inferior Parietal Lobule, the Inferior Frontal Gyrus, and the premotor cortex [47].

Indeed, treatment with AO is essentially based on the principle that “imitation” of movement implies motor observation, motor imagery, and actual execution of movements. Patients are requested to observe and imitate specific actions in order to restore the structures normally activated in the actual execution of those actions [6].

It has been proposed that this mechanism linking observation and action forms the basis by which we understand the actions of others: by mapping the representation of observed actions onto motor systems, observers gain knowledge of those actions by “internally” executing them [48]. From that idea, it has been widely demonstrated that the system linking observation and action can facilitate motor learning [49].

Several studies have consistently shown that AO is an effective way to learn or enhance the performance of a specific motor skill. In a seminal study, participants (required to perform a reaching task in a novel environment) performed better after observing a video depicting a person learning to reach in the same novel environment, than participants who observed the same movement in a different environment [49]. Bove and coworkers [50] showed that the observation of repetitive finger opposition movements at a frequency different from the spontaneous tempo induced changes that closely resembled the observed rhythms and that were long-lasting. Notably, the observation-execution interval had a significant effect on learning: the larger the interval between observation and the first movement execution was, the weaker the effect on the rate of execution of finger movements was. Indeed, it has been proposed that the motor memory of behavioural aspects of an observed rhythmical action can be formed only when movements are promptly executed after video observation [51]. For instance, when AO and physical practice were applied simultaneously it was shown that this combination was more effective to induce both plastic changes in M1 and motor performance improvements than physical practice and AO alone [52–54].

It is postulated that the cortical regions that underlie active motor learning also play a role in motor learning induced by observation. Indeed, passive observation of motor actions induces cortical activity in the primary motor cortex (M1) of the onlooker, which could potentially contribute to motor learning [45]. This facilitation during action observation has been consistently documented and appears to be muscle dependent rather than direction dependent, temporally coupled with the observed action, causally linked to activity in premotor cortex, and dynamically modulated. Recently it has been showed that 30 minutes of repeated thumb movement observation induced neuroplastic changes (LTP, long-term potentiation) in the primary motor cortex, similar to what is seen after physical practice [55]. This result provided some indication as to the underlying neurophysiological mechanism related to the behavioural gains achieved through action observation and suggested that an extended period of action observation may be sufficient to induce LTP in the primary motor cortex.

## 5. Action Observation in Parkinson's Disease

Although the MNS is present in healthy humans, it is still unclear whether it is efficiently working also in PD.

Two studies in PD patients implanted for DBS [56, 57] showed that AO was accompanied by bilateral reduction of the beta oscillatory activity in the STN and of cortico-STN coherence. The occurrence of changes that mimic those observed during actual movement (including the medication effect) suggests that the MNS is reflected in the basal ganglia activity and that it is operating also in PD patients. Further, it has been proposed that the STN might be involved in inhibiting the tendency to carry out the observed action [58].

An original study [59] investigated the effect of viewing action-relevant stimuli (object or finger movements) on reaction-time responses of healthy subjects and PD patients. Both groups produced faster responses when the observed movement matched the direction of their response, but PD subjects lacked specificity for finger movements. Tremblay et al. [21] showed in PD that MEP amplitudes increased during active imitation but not during observation. However, training with Wii Fit was able to improve corticomotor excitability during observation [60]. Castiello et al. [61] made a kinematic analysis of grasping movements after watching a model performing the same movement. PD patients showed AO-related facilitation only when the model was a Parkinsonian subject thus postulating an impaired effectiveness of AO due to damaged basal ganglia function. The latter studies, therefore, would suggest abnormal AO in PD.

On the contrary, Albert et al. [62] using a movement interference task (horizontal/vertical arm or dot movements) found no difference between healthy controls and PD patients (in the “off” condition) thus suggesting that AO system is normally effective in PD. In addition, we recently demonstrated [63] that a single session of AO could reduce bradykinesia of finger movements in PD by improving spontaneous pace. Such effect was still present 45 minutes later only in the “on” condition thus suggesting that the dopaminergic state influences AO ability in PD.

Altogether, available evidences suggest that AO can modify the speed and accuracy of actions in PD, though it is not clear how PD can affect “imitation.”

Several studies investigated treatment with AO for motor rehabilitation of subacute and chronic stroke [53, 64, 65]. On the other hand, very few evidences are available for rehabilitation of patients with PD (see Table 1).

We investigated [66] whether AO, combined with practicing the observed actions, was able to reduce Freezing of Gait (FOG) episodes in PD. Twenty patients entered a single-blind trial and underwent identical physical therapy training but were randomly assigned to the experimental (watching video clips showing specific strategies to circumvent FOG episodes) or control (watching video clips of static different landscapes) groups. The FOG Questionnaire score and the number of FOG episodes were significantly reduced in both groups after the training period, but at follow-up examination (4 weeks after the end of the intervention), a significant reduction in the number of FOG episodes was observed only in the experimental group. This study suggested that AO has a positive additional effect on recovery of walking ability in PD patients.

A pilot RC study investigated the effectiveness of rehabilitative treatment with AO in 15 (Hoehn and Yahr: 2-3) subjects with PD [67]. Individuals in the case group improved significantly more than controls on the UPDRS and the Functional Independence Measure (FIM) scale.

## 6. Conclusions

PD is thought to reflect the dysfunction of circuits interconnecting frontal cortical areas and basal ganglia as a result of the degeneration of the nigrostriatal pathway. Although the pathophysiological mechanisms underlying motor impairment are still uncertain, neurophysiological and neuroimaging studies have been consistent with a deficit in the cortical network subserving movement preparation which translates clinically into cardinal symptoms associated with slowing of motor executions (bradykinesia) and difficulties in action initiation (akinesia).

The dysfunction of the motor cortical network in PD is witnessed by the reduced activation in areas such as the supplementary motor area (SMA) and the primary motor cortex, during performance of motor tasks. However, a compensatory cortical reorganization can be achieved by modulating cortical plasticity through peripheral feedback and sensorimotor integration. Such compensatory reorganization underlies the potential mechanism of rehabilitation interventions.

MI and AO are novel, physiologically well grounded, approaches in neurorehabilitation. Both have the potential to be applied in the rehabilitation of people with PD, though with some limitations. Further research and large, well-designed, RC trials are required to definitely support their efficacy. In addition, it is likely that action representation can be potentiated by concomitant approaches such as cueing [32] or proprioceptive stimulation [68]. It should also be pointed out that although MI and AO are likely to partially share some common mechanisms they cannot be considered interchangeable [6]. MI is more demanding than AO depending on the individuals' capacity to imagine themselves performing specific actions. Further, the correct mental training during MI is difficult to be verified by the therapist. Treatment with AO, therefore, is simpler and more easily to be applied, though a number of details (time and intensity of training, first- or third-person presentation, and type of actions) need to be defined.

## Figures and Tables

**Table 1:** Summary table of studies on rehabilitation with “motor imagery” or “action observation” in Parkinson's disease.

Citation	Groups	Age (years)	Duration (years) H&Y	Type of intervention	Dose of intervention (m/d/w)	Results	FU
Tamir et al. (2007) [39]	Exper. = 12 PD	67.4 ± 9.7	7.4 ± 3.1 2.29 ± 0.4	Combination of imagery + PT	>60/2/12	Significant improvements for the Exper. group in TUG time (decrease 2.5 sec.), getting up from supine (decrease 1.5 sec.), and 360-degree turn. Significant improvement in UPDRS mental section (from 2.1 to 1.2 points).	No
	Control = 11 PD	67.4 ± 9.1	7.8 ± 4.5 2.31 ± 0.4	Only PT		No parallel changes in the control group.	

Braun et al. (2011) [40]	Exper. = 25 PD	70.0 ± 8.0	5.2 ± 5.0 Most < 3	PT + imagery	60/1/6	No significant differences between the groups. General trend in favour of Exper. group.	No

Ajimsha et al. (2014) [41]	Exper. = 32 PD Control = 33 PD	61.4 ± 2.6 60.8 ± 2.1	3.0 ± 0.6 2-3 3.1 ± 0.5 2-3	Autogenic training PT	60/5/8	Significantly greater improvement of UPDRS motor section in the Exper. group after training (51.78% versus 35.24%) and at FU (30.82% versus 21.42%).	12 weeks

Pelosin et al. (2010) [66]	Exper. = 10 PD with FOG Control = 10 PD with FOG	68.8 ± 4.1 70.2 ± 6.8	11.6 ± 4.9 2.1 ± 0.3 9.5 ± 3.7 2.2 ± 0.3	Action observation + PT Landscape observation + PT	60/3/4	Significant improvement in both groups of motor performance (UPDRS-III, TUG, 10 M-WT) and quality of life (PDQ-39) after training and at FU. FOG-Q and number of FOG episodes significantly reduced in both groups after training, but only in Exper. group at FU.	4 weeks

Buccino et al. (2011) [67]	Exper. = 7 PD Control = 8 PD	68 (59–80) 73.5 (67.5–76.5)	7 (5–19) 3 (2.5–4) 9 (5.5–13.5) 1.7 (1.5–2.3)	Action observation + PT PT only	—	Significantly greater improvements in Exper. group for UPDRS and FIM scores.	No

PD = Parkinson's disease, H&Y = Hoehn and Yahr stage, PT = physical therapy, TUG = time-up-and-go test, UPDRS = Unified Parkinson's Disease Rating Scale, FOG = Freezing of Gait, 10 M-WT = 10-meter walking test, FOG-Q = Freezing of Gait Questionnaire, FIM = Functional Independence Measure Scale, FU = follow-up, and PDQ-39 = Parkinson's Disease Questionnaire.

## References

[1] Tomlinson C. L., Patel S., Meek C. (2012). Physiotherapy versus placebo or no intervention in Parkinson's disease. *Cochrane Database of Systematic Reviews*.

[2] Tomlinson C. L., Herd C. P., Clarke C. E. (2014). Physiotherapy for Parkinson's disease: a comparison of techniques. *Cochrane Database of Systematic Reviews*.

[3] Baker K., Rochester L., Nieuwboer A. (2007). The immediate effect of attentional, auditory, and a combined cue strategy on gait during single and dual tasks in Parkinson's disease. *Archives of Physical Medicine and Rehabilitation*.

[4] Mulder T. (2007). Motor imagery and action observation: cognitive tools for rehabilitation. *Journal of Neural Transmission*.

[5] Malouin F., Jackson P. L., Richards C. L. (2013). Towards the integration of mental practice in rehabilitation programs. A critical review. *Frontiers in Human Neuroscience*.

[6] Buccino G. (2014). Action observation treatment: a novel tool in neurorehabilitation. *Philosophical Transactions of the Royal Society of London Series B*.

[7] Decety J. (1996). The neurophysiological basis of motor imagery. *Behavioural Brain Research*.

[8] Crammond D. J. (1997). Motor imagery: never in your wildest dream. *Trends in Neurosciences*.

[9] Jeannerod M., Decety J. (1995). Mental motor imagery: a window into the representational stages of action. *Current Opinion in Neurobiology*.

[10] Decety J. (1996). Do imagined and executed actions share the same neural substrate?. *Cognitive Brain Research*.

[11] Abbruzzese G., Trompetto C., Schieppati M. (1996). The excitability of the human motor cortex increases during execution and mental imagination of sequential but not repetitive finger movements. *Experimental Brain Research*.

[12] Stephan K. M., Fink G. R., Passingham R. E. (1995). Functional anatomy of the mental representation of upper extremity movements in healthy subjects. *Journal of Neurophysiology*.

[13] Deiber M.-P., Ibañez V., Honda M., Sadato N., Raman R., Hallett M. (1998). Cerebral processes related to visuomotor imagery and generation of simple finger movements studied with positron emission tomography. *NeuroImage*.

[14] Papaxanthis C., Pozzo T., Skoura X., Schieppati M. (2002). Does order and timing in performance of imagined and actual movements affect the motor imagery process? The duration of walking and writing task. *Behavioural Brain Research*.

[15] Decety J., Jeannerod M., Durozard D., Baverel G. (1993). Central activation of autonomic effectors during mental simulation of motor actions in man. *Journal of Physiology*.

[16] Jeannerod M. (2001). Neural simulation of action: a unifying mechanism for motor cognition. *NeuroImage*.

[17] Pascual-Leone A., Nguyet D., Cohen L. G., Brasil-Neto J. P., Cammarota A., Hallett M. (1995). Modulation of muscle responses evoked by transcranial magnetic stimulation during the acquisition of new fine motor skills. *Journal of Neurophysiology*.

[18] Louis M., Guillot A., Maton S., Doyon J., Collet C. (2008). Effect of imagined movement speed on subsequent motor performance. *Journal of Motor Behavior*.

[19] Avanzino L., Giannini A., Tacchino A., Pelosin E., Ruggeri P., Bove M. (2009). Motor imagery influences the execution of repetitive finger opposition movements. *Neuroscience Letters*.

[20] Avanzino L., Gueugneau N., Bisio A., Ruggeri P., Papaxanthis C., Bove M. (2015). Motor cortical plasticity induced by motor learning through mental practice. *Frontiers in Behavioral Neuroscience*.

[21] Tremblay F., Léonard G., Tremblay L. (2008). Corticomotor facilitation associated with observation and imagery of hand actions is impaired in Parkinson's disease. *Experimental Brain Research*.

[22] Cunnington R., Iansek R., Johnson K. A., Bradshaw J. L. (1997). Movement-related potentials in Parkinson's disease: motor imagery and movement preparation. *Brain*.

[23] Cunnington R., Egan G. F., O'Sullivan J. D., Hughes A. J., Bradshaw J. L., Colebatch J. G. (2001). Motor imagery in Parkinson's disease: a PET study. *Movement Disorders*.

[24] Thobois S., Dominey P. F., Decety J. (2000). Motor imagery in normal subjects and in asymmetrical Parkinson's disease: a PET study. *Neurology*.

[25] Helmich R. C., de Lange F. P., Bloem B. R., Toni I. (2007). Cerebral compensation during motor imagery in Parkinson's disease. *Neuropsychologia*.

[26] van Nuenen B. F. L., Helmich R. C., Buenen N., van de Warrenburg B. P. C., Bloem B. R., Toni I. (2012). Compensatory activity in the extrastriate body area of Parkinson's disease patients. *The Journal of Neuroscience*.

[27] Leiguarda R., Cerquetti D., Tenca E., Merello M. (2009). Globus pallidus internus firing rate modification after motor-imagination in three Parkinson's disease patients. *Journal of Neural Transmission*.

[28] Kühn A. A., Doyle L., Pogosyan A. (2006). Modulation of beta oscillations in the subthalamic area during motor imagery in Parkinson's disease. *Brain*.

[29] Thobois S., Dominey P., Fraix V. (2002). Effects of subthalamic nucleus stimulation on actual and imagined movement in Parkinson's disease: a PET study. *Journal of Neurology*.

[30] Di Rienzo F., Collet C., Hoyek N., Guillot A. (2014). Impact of neurologic deficits on motor imagery: a systematic review of clinical evaluations. *Neuropsychology Review*.

[31] Heremans E., Feys P., Nieuwboer A. (2011). Motor imagery ability in patients with early- and mid-stage Parkinson disease. *Neurorehabilitation and Neural Repair*.

[32] Heremans E., Nieuwboer A., Feys P. (2012). External cueing improves motor imagery quality in patients with Parkinson disease. *Neurorehabilitation and Neural Repair*.

[33] Peterson D. S., Pickett K. A., Earhart G. M. (2012). Effects of levodopa on vividness of motor imagery in Parkinson disease. *Journal of Parkinson's Disease*.

[34] Maillet A., Thobois S., Fraix V. (2015). Neural substrates of levodopa-responsive gait disorders and freezing in advanced Parkinson's disease: a kinesthetic imagery approach. *Human Brain Mapping*.

[35] Avanzino L., Pelosin E., Martino D., Abbruzzese G. (2013). Motor timing deficits in sequential movements in Parkinson disease are related to action planning: a motor imagery study. *PLoS ONE*.

[36] Conson M., Trojano L., Vitale C. (2014). The role of embodied simulation in mental transformation of whole-body images: evidence from Parkinson's disease. *Human Movement Science*.

[37] Peterson D. S., Pickett K. A., Duncan R. P., Perlmutter J. S., Earhart G. M. (2014). Brain activity during complex imagined gait tasks in Parkinson disease. *Clinical Neurophysiology*.

[38] Snijders A. H., Leunissen I., Bakker M. (2011). Gait-related cerebral alterations in patients with Parkinson's disease with freezing of gait. *Brain*.

[39] Tamir R., Dickstein R., Huberman M. (2007). Integration of motor imagery and physical practice in group treatment applied to subjects with Parkinson's disease. *Neurorehabilitation and Neural Repair*.

[40] Braun S., Beurskens A., Kleynen M., Schols J., Wade D. (2011). Rehabilitation with mental practice has similar effects on mobility as rehabilitation with relaxation in people with Parkinson's disease: a multicentre randomised trial. *Journal of Physiotherapy*.

[41] Ajimsha M. S., Majeed N. A., Chinnavan E., Thulasyammal R. P. (2014). Effectiveness of autogenic training in improving motor performances in Parkinson's disease. *Complementary Therapies in Medicine*.

[42] Gallese V., Fadiga L., Fogassi L., Rizzolatti G. (1996). Action recognition in the premotor cortex. *Brain*.

[43] Rizzolatti G., Fadiga L., Gallese V., Fogassi L. (1996). Premotor cortex and the recognition of motor actions. *Cognitive Brain Research*.

[44] Fabbri-Destro M., Rizzolatti G. (2008). Mirror neurons and mirror systems in monkeys and humans. *Physiology*.

[45] Fadiga L., Fogassi L., Pavesi G., Rizzolatti G. (1995). Motor facilitation during action observation: a magnetic stimulation study. *Journal of Neurophysiology*.

[46] Hari R., Forss N., Avikainen S., Kirveskari E., Salenius S., Rizzolatti G. (1998). Activation of human primary motor cortex during action observation: a neuromagnetic study. *Proceedings of the National Academy of Sciences of the United States of America*.

[47] Ertelt D., Small S., Solodkin A. (2007). Action observation has a positive impact on rehabilitation of motor deficits after stroke. *NeuroImage*.

[48] Rizzolatti G., Fogassi L., Gallese V. (2001). Neurophysiological mechanisms underlying the understanding and imitation of action. *Nature Reviews Neuroscience*.

[49] Mattar A. A. G., Gribble P. L. (2005). Motor learning by observing. *Neuron*.

[50] Bove M., Tacchino A., Pelosin E., Moisello C., Abbruzzese G., Ghilardi M. F. (2009). Spontaneous movement tempo is influenced by observation of rhythmical actions. *Brain Research Bulletin*.

[51] Zhang X., de Beukelaar T. T., Possel J. (2011). Movement observation improves early consolidation of motor memory. *The Journal of Neuroscience*.

[52] Celnik P., Stefan K., Hummel F., Duque J., Classen J., Cohen L. G. (2006). Encoding a motor memory in the older adult by action observation. *NeuroImage*.

[53] Celnik P., Webster B., Glasser D. M., Cohen L. G. (2008). Effects of action observation on physical training after stroke. *Stroke*.

[54] Stefan K., Classen J., Celnik P., Cohen L. G. (2008). Concurrent action observation modulates practice-induced motor memory formation. *European Journal of Neuroscience*.

[55] Lepage J.-F., Morin-Moncet O., Beaulé V., de Beaumont L., Champoux F., Théoret H. (2012). Occlusion of LTP-like plasticity in human primary motor cortex by action observation. *PLoS ONE*.

[56] Marceglia S., Fiorio M., Foffani G. (2009). Modulation of beta oscillations in the subthalamic area during action observation in Parkinson's disease. *Neuroscience*.

[57] Alegre M., Rodríguez-Oroz M. C., Valencia M. (2010). Changes in subthalamic activity during movement observation in Parkinson's disease: is the mirror system mirrored in the basal ganglia?. *Clinical Neurophysiology*.

[58] Alegre M., Guridi J., Artieda J. (2011). The mirror system, theory of mind and Parkinson's disease. *Journal of the Neurological Sciences*.

[59] Poliakoff E., Galpin A., Dick J., Moore P., Tipper S. P. (2007). The effect of viewing graspable objects and actions in Parkinson's disease. *NeuroReport*.

[60] Esculier J.-F., Vaudrin J., Tremblay L. E. (2014). Corticomotor excitability in parkinson's disease during observation, imagery and imitation of action: effects of rehabilitation using wii fit and comparison to healthy controls. *Journal of Parkinson's Disease*.

[61] Castiello U., Ansuini C., Bulgheroni M., Scaravilli T., Nicoletti R. (2009). Visuomotor priming effects in Parkinson's disease patients depend on the match between the observed and the executed action. *Neuropsychologia*.

[62] Albert N., Peiris Y., Cohen G., Miall R., Praamstra P. (2010). Interference effects from observed movement in Parkinson's disease. *Journal of Motor Behavior*.

[63] Pelosin E., Bove M., Ruggeri P., Avanzino L., Abbruzzese G. (2013). Reduction of bradykinesia of finger movements by a single session of action observation in Parkinson disease. *Neurorehabilitation and Neural Repair*.

[64] Sale P., Franceschini M. (2012). Action observation and mirror neuron network: a tool for motor stroke rehabilitation. *European Journal of Physical and Rehabilitation Medicine*.

[65] Sugg K., Müller S., Winstein C., Hathorn D., Dempsey A. (2015). Does action observation training with immediate physical practice improve hemiparetic upper-limb function in chronic stroke?. *Neurorehabilitation and Neural Repair*.

[66] Pelosin E., Avanzino L., Bove M., Stramesi P., Nieuwboer A., Abbruzzese G. (2010). Action observation improves freezing of gait in patients with Parkinson's disease. *Neurorehabilitation and Neural Repair*.

[67] Buccino G., Gatti R., Giusti M. C. (2011). Action observation treatment improves autonomy in daily activities in Parkinson's disease patients: results from a pilot study. *Movement Disorders*.

[68] Bisio A., Avanzino L., Gueugneau N., Pozzo T., Ruggeri P., Bove M. (2015). Observing and perceiving: a combined approach to induce plasticity in human motor cortex. *Clinical Neurophysiology*.

